# Skin autofluorescence predicts new cardiovascular disease and mortality in people with type 2 diabetes

**DOI:** 10.1186/s12902-020-00676-4

**Published:** 2021-01-12

**Authors:** Henderikus E. Boersma, Robert P. van Waateringe, Melanie M. van der Klauw, Reindert Graaff, Andrew D. Paterson, Andries J. Smit, Bruce H. R. Wolffenbuttel

**Affiliations:** 1 Department of Endocrinology, University of Groningen, University Medical Center Groningen, P.O. Box 30001, HPC AA31, Groningen, RB 9700 The Netherlands; 2 Department of Internal Medicine, University of Groningen, University Medical Center Groningen, Groningen, The Netherlands; 3 Program in Genetics and Genome Biology, Hospital for Sick Children, Toronto, ON Canada

**Keywords:** Ageing, Cardiovascular disease, Diabetes, Mortality, Prediction, Skin autofluorescence

## Abstract

**Background:**

Skin autofluorescence (SAF) is a non-invasive marker of tissue accumulation of advanced glycation endproducts (AGE). Recently, we demonstrated in the general population that elevated SAF levels predict the development of type 2 diabetes (T2D), cardiovascular disease (CVD) and mortality. We evaluated whether elevated SAF may predict the development of CVD and mortality in individuals with T2D.

**Methods:**

We included 2349 people with T2D, available baseline SAF measurements (measured with the AGE reader) and follow-up data from the Lifelines Cohort Study. Of them, 2071 had no clinical CVD at baseline. 60% were already diagnosed with diabetes (median duration 5, IQR 2–9 years), while 40% were detected during the baseline examination by elevated fasting blood glucose ≥7.0 mmol/l) and/or HbA1c ≥6.5% (48 mmol/mol).

**Results:**

Mean (±SD) age was 57 ± 12 yrs., BMI 30.2 ± 5.4 kg/m^2^. 11% of participants with known T2D were treated with diet, the others used oral glucose-lowering medication, with or without insulin; 6% was using insulin alone. Participants with known T2D had higher SAF than those with newly-detected T2D (SAF Z-score 0.56 ± 0.99 vs 0.34 ± 0.89 AU, *p* < 0.001), which reflects a longer duration of hyperglycaemia in the former group. Participants with existing CVD and T2D had the highest SAF Z-score: 0.78 ± 1.25 AU. During a median follow-up of 3.7 yrs., 195 (7.6%) developed an atherosclerotic CVD event, while 137 (5.4%) died. SAF was strongly associated with the combined outcome of a new CVD event or mortality (OR 2.59, 95% CI 2.10–3.20, *p* < 0.001), as well as incidence of CVD (OR 2.05, 95% CI 1.61–2.61, *p* < 0.001) and death (OR 2.98, 2.25–3.94, *p* < 0.001) as a single outcome. In multivariable analysis for the combined endpoint, SAF retained its significance when sex, systolic blood pressure, HbA1c, total cholesterol, eGFR, as well as antihypertensive and statin medication were included. In a similar multivariable model, SAF was independently associated with mortality as a single outcome, but not with incident CVD.

**Conclusions:**

Measuring SAF can assist in prediction of incident cardiovascular disease and mortality in individuals with T2D. SAF showed a stronger association with future CVD events and mortality than cholesterol or blood pressure levels.

**Supplementary Information:**

The online version contains supplementary material available at 10.1186/s12902-020-00676-4.

## Background

Cardiovascular complications are an important cause of diabetes-related morbidity and excess mortality [1–3]. Various risk factors, such as obesity, and blood pressure (BP) levels, lipids and glycaemic parameters predict the development of cardiovascular disease (CVD). In the past, a variety of risk scores has been developed to assist in adequate disease prediction [4–7].

In the pathophysiology of diabetes-related complications, the role of the accumulation of advanced glycation endproducts (AGEs) has been well established. These AGEs are formed in a complex biochemical process, by glycation of proteins and lipids in the classic Maillard ‘browning’ reaction, but also by the interaction of amino groups of proteins with α-dicarbonyl compounds like as glyoxal, methylglyoxal and 3-deoxyglucosone [8–10]. Crosslinking of AGEs and tissue proteins in the body, may cause for instance an increase of vascular stiffness, elevated blood pressure and limited joint mobility [11, 12]. Binding of circulating AGEs to specific receptors (i.e. the receptors for AGEs [RAGE]) and subsequent uptake in arterial walls may play an important role in the development and progression of atherosclerosis [13, 14].

The accumulation of AGEs can be evaluated by measuring skin autofluorescence (SAF) [15]. This non-invasive method has been described and validated extensively, and it has been shown that SAF strongly correlates with the levels of AGEs in skin biopsies [16]. Earlier studies have shown that SAF is higher in individuals with type 2 diabetes compared with healthy individuals [17, 18], and is associated with the development of cardiovascular complications and mortality in these patients [17, 19–21]. An earlier study by our group in the general population has shown that SAF is a strong predictor of type 2 diabetes and cardiovascular disease, as well as mortality, independent of several classic risk factors [22].

The aim of the present study was to evaluate whether SAF is able to predict the development of cardiovascular disease and mortality in individuals with type 2 diabetes, who participated in the Lifelines Cohort Study.

## Methods

### Participants

We evaluated participants with type 2 diabetes who participated between 2007 and 2013 in the Lifelines Cohort Study. Lifelines is a large population-based study of residents living in the northern provinces of the Netherlands [23]. At baseline evaluation, both extensive questionnaire and physical examination data were collected [24]. The study was approved by the Medical Ethics Review Committee of the University Medical Center Groningen. All participants provided written informed consent.

Presence of type 2 diabetes was self-reported or based on the use of blood-glucose-lowering medication (oral agents and/or insulin), or fasting blood glucose ≥7.0 mmol/l and/or HbA1c ≥ 6.5% (48 mmol/mol) at laboratory evaluation. We did not include participants who, at baseline, reported type 1 diabetes (*n* = 366) or MODY (*n* = 9), or previously having gestational diabetes (*n* = 266). In 2554 out of 4992 people with type 2 diabetes, validated baseline SAF measurement were available (Supplemental Fig. 1). Participants with available SAF measurements did not differ in sex ratio, age, glucose and HbA_1c_ from those without SAF measurements. Follow-up data were available for 2349 individuals. Of these, 1863 completed the follow-up questionnaires and additional laboratory testing between 2014 and 2018. Of the remaining 486 individuals only interim questionnaire results were available. Duration of follow-up was 3.7 (range 0.5–10) years and comprised 8637 participant-years. In the 1863 participants with complete questionnaire and laboratory data, median follow-up was 4.0 years.

### Clinical examination

Information on medical history, health status and lifestyle including smoking habits were collected using self-administered questionnaires as published previously (22). Smoking status was classified into never, former or current smoking. The use of medication was verified using the ATC (Anatomical Therapeutic Chemical) Classification System by a research assistant at the baseline investigation only. Weight, height and waist circumference were measured while participants were wearing light clothing and no shoes. Blood pressure and heart rate were measured using an automated Dinamap monitor (GE Healthcare, Freiburg, Germany), for a total of 10 measurements every 10 min, and BP and heart rate were determined as the average of the last three readings.

### Skin autofluorescence

At baseline, SAF, expressed in arbitrary units, was measured at the forearm using an AGE Reader (Diagnoptics Technologies, Groningen, the Netherlands), as described previously [18, 22, 25]. We calculated SAF Z-scores (adjusted for age) based on the total Lifelines population, separately in men and women.

### Biochemical measurements

Blood was drawn between 8 and 10 a.m. while participants were in the fasting state. For the current study, baseline biochemical measurements used for analysis were performed the same day. HbA_1c_ was measured in EDTA-anticoagulated blood on a Cobas Integra 800 CTS analyser (Roche, The Netherlands) with a NGSP (National Glycohemoglobin Standardized Program) certified turbidimetric immunoassay. Blood glucose was measured with a hexokinase method. Serum concentrations of creatinine and lipids (total-, HDL- and LDL-cholesterol, and triacylglycerol were measured on a Roche Modular P chemistry analyser (Roche, Basel, Switzerland) [22]. Estimated (e) GFR was calculated using the CKD-EPI (Chronic Kidney Disease Epidemiology Collaboration) formula [26].

### Calculations, definitions and statistical analyses

Baseline as well as new events of cardiovascular disease were defined as a previous or an incident of myocardial infarction, transient ischaemic attack (TIA), cerebrovascular accident (CVA), intermittent claudication or therapeutic intervention including percutaneous transluminal coronary angioplasty (PTCA) with or without stent, coronary artery bypass grafting (CABG) or peripheral vascular surgery. All clinical outcomes were self-reported. Vital status was confirmed with the municipal administration. This database does not contain information on the cause of death. For all age groups, the incidence of cardiovascular disease and all-cause mortality was calculated for all predefined age decades separately and as a composite outcome.

Normally-distributed data are presented as mean ± SD, otherwise median and interquartile range (IQR) was used. The difference between groups were evaluated with analysis of variance (ANOVA) or the Mann–Whitney *U* test. The *χ*^2^ test was used to analyse categorical variables. Univariable and multivariable logistic regression analysis was used to evaluate the association between SAF and the composite outcome of cardiovascular disease and mortality, with adjustment for the most important baseline variables. A second model was constructed in which only significant variables were included. We did this for the entire type 2 diabetes population, and separately for those individuals with diabetes without clinically manifest cardiovascular disease at baseline. Analyses were conducted with PASW Statistics (Version 23, IBM, Armonk, NY, USA). *P*-values < 0.05 were considered statistically significant.

## Results

### Baseline characteristics

**Fig. 1:**
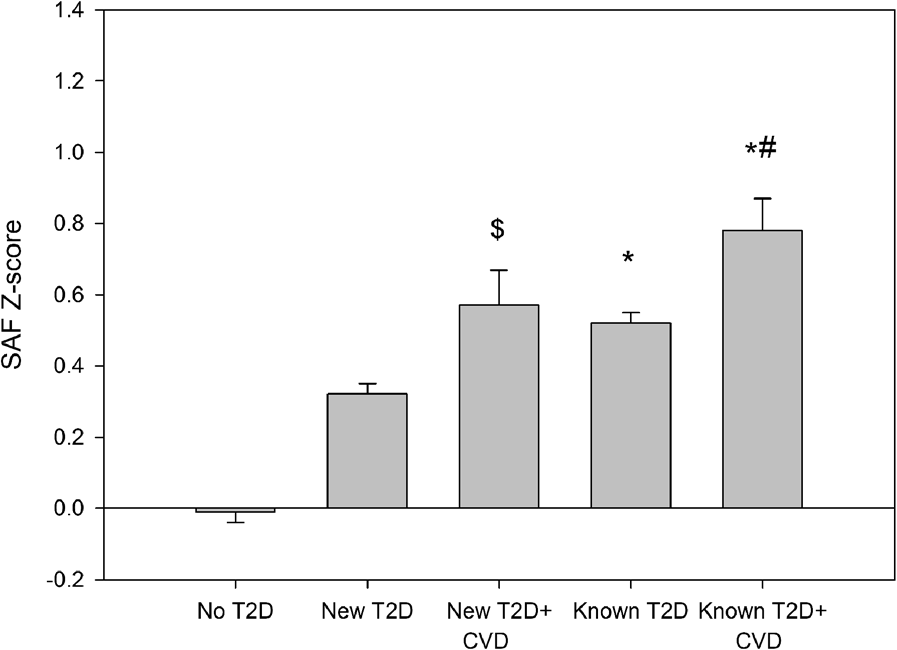
Z-score of skin autofluorescence in relationship to presence of type 2 diabetes and cardiovascular disease. Data are presented as mean ± SEM. For comparison, age-corrected SAF scores are compared with Lifelines participants without diabetes (‘No T2D’). * denotes *p* < 0.001 versus people with New T2D; $ *p* = 0.009 vs New T2D; # *p* < 0.001 vs Known T2D. All are *p* < 0.001 vs No T2D

At baseline, 1318 subjects reported a previous diagnosis of type 2 diabetes (‘known type 2 diabetes’), and their estimated duration of diabetes in these participants was 5 (IQR 2–9) years. In addition, 1031 participants were found to have elevated fasting blood glucose and/or HbA1c at laboratory evaluation, and those individuals were considered as new type 2 diabetes. They were notified of the findings and the laboratory results were reported to the general practitioner. Only 11% of the participants with known type 2 diabetes were treated with diet, 83% was treated with oral glucose-lowering agents, with or without insulin, while 6% was using insulin alone. Individuals with known type 2 diabetes were older than those with new type 2 diabetes (58.7 ± 10.8 years vs 55.0 ± 12.0 years, *p* < 0.001). They also had a higher BMI and HbA_1c_ (all *p* < 0.01, Table 1), and a larger percentage of them were reporting the use of blood-pressure-lowering medication and/or statins, and they had lower total and LDL-cholesterol. Participants with newly-detected type 2 diabetes had lower SAF levels than those with known diabetes (SAF Z-score 0.34 ± 0.89 vs 0.56 ± 0.99 AU, *p* < 0.001, Table 1). In Fig. 1, we show the age-corrected SAF levels for participants with and without cardiovascular disease, in comparison with Lifelines participants without diabetes. SAF Z-scores were the highest in participants with both existing diabetes and cardiovascular disease: 0.78 ± 1.10 AU (Fig. 1).

**Table 1:** Clinical characteristics of the study population at baseline

Characteristic	New T2D	Known T2D	*P*-value
Sex (*n*; male/female)	569/462	656/662	0.009
Age (years)	55.0 ± 12.0	58.7 ± 10.8	< 0.001
BMI (kg/m^2^)	29.9 ± 5.2	30.5 ± 5.5	0.009
Waist (cm)	103 ± 13	104 ± 14	0.009
Systolic BP (mmHg)	136 ± 17	135 ± 17	0.103
Diastolic BP (mmHg)	78 ± 10	75 ± 9	< 0.001
Heart rate (bpm)	73 ± 12	74 ± 12	0.827
Creatinine (μmol/l)	75 ± 16	74 ± 17	0.052
eGFR (ml/min/1.73m^2^)	90 ± 16	88 ± 17	0.003
Total cholesterol (mmol/l)	5.3 ± 1.2	4.5 ± 1.0	< 0.001
HDL-cholesterol (mmol/l)	1.26 ± 0.36	1.27 ± 0.36	0.433
LDL-cholesterol (mmol/l)	3.4 ± 1.0	2.7 ± 0.9	< 0.001
Triacylglycerol (mmol/l)	1.88 ± 1.30	1.70 ± 1.11	< 0.001
Glucose (mmol/l)	7.6 ± 2.3	7.6 ± 2.1	0.747
HbA_1c_ (mmol/mol)	49 ± 14	52 ± 11	< 0.001
HbA_1c_ (%)	6.7 ± 1.2	6.9 ± 1.0	< 0.001
Current smoking (%)	23.7	14.4	< 0.001
Former smoking (%)	42.5	51.1	< 0.001
% w. BP-lowering therapy	35.7	63.4	< 0.001
% w. statins	20.1	63.0	< 0.001
Skin autofluorescence (AU)	2.25 ± 0.50	2.42 ± 0.54	< 0.001
SAF *z* score	0.34 ± 0.89	0.56 ± 0.99	< 0.001

Data are presented as mean ± SD, number or %

In total, 195 individuals (7.6%) had developed a new CVD event at follow-up. Those with new CVD events were significantly older at baseline (62 ± 11 vs 56 ± 12 yrs., *p* < 0.01), and had a lower eGFR (85 ± 16 vs 90 ± 16 ml/min/1.73m^2^, *p* < 0.001) compared to those without new CVD events. Levels of blood pressure, serum lipids and glycaemic parameters were not significantly different. Incidence of cardiovascular disease was higher with increasing age, and was between 9.7 and 33% in the highest age groups (Suppl. Fig. 2). Mean SAF Z-score at baseline was 0.70 ± 1.11 among the participants who developed a new CVD event vs 0.44 ± 0.93 in those who did not (*p =* 0.001).

Death occurred in 137 individuals (5.4%). As expected, mortality was higher in older participants, and was between 5.5 and 52% in the highest age groups (Suppl. Fig. 2). Participants who deceased were older at baseline (67 ± 12 vs 56 ± 11 yrs., *p* < 0.001), had lower eGFR (79 ± 19 vs 90 ± 16 ml/min/1.73m^2^, *p* < 0.001), and more often renal impairment (eGFR < 60 ml/min/1.73m^2^, 18.5% vs 4.1%, *p* < 0.001). They also had higher SAF levels than individuals who remained alive: SAF Z-score was 0.81 ± 1.06 in those who died vs 0.44 ± 0.94 in those who remained alive (*p* < 0.001).

Existing cardiovascular disease at baseline was strongly associated with outcome: incidence of new CVD events and death was significantly higher in participants with diabetes and CVD at baseline vs those with diabetes only, both in those with known and newly-detected diabetes (Table 2). Similarly, incidence of cardiovascular disease but not mortality was higher in those with known diabetes compared to those whose diabetes was detected at the baseline screening.

**Table 2:** Incidence of CVD and death (percentage of participants) according to baseline diabetes and CVD status

	New T2D, no CVD (*n* = 933)	New T2D, CVD (*n* = 98)	*P*-value	Known T2D, no CVD (*n* = 1138)	Known T2D, CVD (*n* = 180)	*P*-value
New CVD events (%)	44 (4.7%)	25 (25.5%)	< 0.001	66 (5.8%)	60 (33.3%)	< 0.001
Death (%)	47 (5.0%)	16 (16.3%)	< 0.001	47 (4.1%)	27 (15.0%)	< 0.001
CVD or death (%)	88 (9.4%)	38 (38.8%)	< 0.001	110 (9.7%)	77 (42.8%)	< 0.001

### Association and prediction

Table 3 summarizes the univariable and multivariable associations between SAF and clinical, biochemical and lifestyle factors and the combined outcome of cardiovascular disease and mortality. Univariable analysis showed that SAF was significantly associated with combined outcome (OR 2.59, 95% CI 2.10–3.20, *p* = 1.3 × 10^− 18^). Also, age, male sex, waist circumference, diastolic BP, heart rate, eGFR, as well as the use of BP-lowering medication and statins, and baseline CVD showed a significant association with the combined outcome. The association of SAF with outcome remained significant after adjusting for systolic BP, serum lipids, eGFR and glycaemic variables (OR 1.41, 95% CI 1.10–1.82, *p* = 0.008). In the multivariable model (Table 3), baseline cardiovascular disease, age, current smoking, SAF and systolic BP showed the strongest association with the combined outcome.

**Table 3:** Univariable and multivariable logistic regression analyses for the composite primary outcome (CVD or death) at a median of 3.7 years’ follow-up

Analysis	n	OR	95% CI	***P***-value
Univariable				
SAF (AU)	2349	2.59	2.10–3.20	1.3 × 10^−18^
Age (years)	2349	1.06	1.05–1.08	4.4 × 10^−25^
Male sex (y/n)	2349	1.49	1.17–1.90	0.001
BMI (kg/m^2^)	2349	1.00	0.97–1.02	0.697
Waist circumference (cm)	2347	1.01	1.00–1.02	0.025
Glucose (mmol/l)	2333	0.99	0.94–1.05	0.859
HbA_1c_ (mmol/mol)	2334	1.01	1.00–1.02	0.092
SBP (mmHg)	2343	1.00	0.99–1.00	0.138
DBP (mmHg)	2343	0.97	0.96–0.99	5.2 × 10^−5^
HR (/min)	2343	0.99	0.98–1.00	0.013
Cholesterol (mmol/l)	2341	0.91	0.82–1.01	0.070
Triacylglycerol (mmol/l)	2341	0.99	0.89–1.09	0.781
eGFR (ml/min/1.73m^2^)	2342	0.97	0.97–0.98	6.0 × 10^−14^
Former smoking (y/n)	2335	1.30	1.02–1.64	0.033
Current smoking (y/n)	2335	1.19	0.89–1.60	0.246
Statin (y/n)	2349	1.44	1.14–1.83	0.002
BP-lowering therapy (y/n)	2349	2.28	1.77–2.94	1.7 × 10^−10^
Baseline CVD (y/n)	2349	6.67	5.04–8.83	2.8 × 10^−40^
Multivariable model 1	2310			
SAF (AU)		1.41	1.10–1.82	0.008
Age (years)		1.05	1.03–1.07	5.9 × 10^−8^
Male sex (y/n)		1.16	0.84–1.62	0.368
BMI (kg/m^2^)		0.96	0.90–1.01	0.126
Waist (cm)		1.02	1.00–1.05	0.031
Glucose (mmol/l)		0.99	0.90–1.08	0.784
HbA1c (mmol/mol)		1.01	1.00–1.03	0.124
SBP (mmHg)		0.99	0.98–1.00	0.051
DBP (mmHg)		0.99	0.97–1.01	0.411
HR (/min)		1.01	1.00–1.02	0.081
Cholesterol (mmol/l)		1.08	0.93–1.25	0.331
Triacylglycerol (mmol/l)		1.01	0.89–1.15	0.848
eGFR (ml/min/1.73m^2^)		1.00	0.99–1.01	0.845
Former smoking (y/n)		0.95	0.69–1.30	0.738
Current smoking (y/n)		1.69	1.13–2.53	0.010
Statin (y/n)		0.72	0.51–1.01	0.054
BP-lowering therapy (y/n)		1.33	0.97–1.82	0.075
Baseline CVD (y/n)		5.25	3.71–7.42	7.4 × 10^−21^
Multivariable model 2	2327			
SAF (AU)		1.48	1.15–1.90	0.002
Age (years)		1.06	1.04–1.07	8.2 × 10^−14^
Waist (cm)		1.02	1.00–1.02	0.005
SBP (mmHg)		0.99	0.98–1.00	0.003
Current smoking (y/n)		1.83	1.31–2.56	4.4 × 10^−4^
Statin (y/n)		0.70	0.53–0.94	0.016
Baseline CVD (y/n)		5.30	3.84–7.33	4.8 × 10^−24^

Baseline risk factors were used to predict the median 3.7 year risk of the composite outcome of new CVD events and death

SAF, age, glucose, HbA_1c_, waist circumference, systolic and diastolic BP, HR, cholesterol, triacylglycerol and eGFR were defined as continuous variables. Male sex, current smoking, statin use, use of BP-lowering therapy and baseline CVD were defined as categorical variables

DBP, diastolic BP; SBP, systolic BP; HR, heart rate; y/n, yes/no

In Supplemental Table 1 we report the association between SAF and the individual outcomes. Univariable analyses showed SAF was significantly associated with mortality (OR 2.98, 95% CI 2.25–3.94, *p* = 2.6 × 10^− 14^), as were age, sex, waist circumference, diastolic BP, eGFR, as well as BP-lowering therapy and baseline cardiovascular disease. This association remained significant after adjusting for all other baseline variables (*p* = 0.013). The multivariable model showed that SAF, age, systolic BP, waist circumference, current smoking, statin use and baseline cardiovascular disease were independently associated with mortality. Comparable, SAF was also associated with new CVD events. In addition to SAF and age, other predictors for new CVD events were male sex, eGFR, diastolic BP and heart rate, as well as the use of statins, BP-lowering medication and baseline cardiovascular disease. SAF was no longer significant with incident cardiovascular disease in the multivariable models, in which age, current smoking and baseline cardiovascular disease showed the strongest association.

As baseline cardiovascular disease is a strong predictor of future CVD events and mortality, we re-calculated our models for the combined outcome of cardiovascular disease and mortality in participants without baseline cardiovascular disease. Again, SAF was strongly associated with the combined outcome, even when adjusted for the other variables age, systolic BP, waist circumference, statin use and current smoking (Supplemental Table 2).

## Discussion

In a previous study in the general population, we have shown that skin autofluorescence is strongly associated with new-onset of type 2 diabetes and cardiovascular disease as well as mortality. This association was independent of cardiovascular risk factors like age, sex, waist circumference, smoking, and glycaemic parameters. In the present study we show that SAF is also significantly and independently associated with the combined outcome of new CVD events and mortality in people with type 2 diabetes.

It has long been known that the formation of AGEs is increased in people with diabetes as a consequence of high glucose levels and oxidative stress [8, 27]. Earlier, we have shown that SAF levels are increased in people with the metabolic syndrome, a cluster of risk factors which is associated with an increased risk of both type 2 diabetes and cardiovascular disease [28]. However, when adjusted for presence of the metabolic syndrome, SAF levels were an independent predictor of new type 2 diabetes in the general population [22]. Our current analyses shows that individuals with newly-detected type 2 diabetes had lower SAF Z-scores than those with known type 2 diabetes, indicating the longer period of exposure to elevated glucose levels in people with longer-standing diabetes. In addition, participants with cardiovascular disease had higher SAF levels, both those with newly-detected and those with known diabetes (Fig. 1). Existing cardiovascular disease was the strongest predictor for future CVD events and mortality. Nevertheless, SAF was associated with this combined outcome independently of factors like existing cardiovascular disease, age, smoking, lipid or BP levels.

SAF was associated with a threefold increased mortality risk, and this association remained highly significant after adjusting for several confounding factors (Supplemental Table 1). This confirms our earlier observations in people without diabetes [22]. In that study a high odds ratio for mortality was also obtained when modelling with the linear method based on the entire population without diabetes or cardiovascular disease. Similarly, SAF was associated with an increased risk of new CVD events. Several earlier reports have described the cross-sectional relationship between SAF and the presence and development of vascular complications in type 2 diabetes. Prospective studies evaluating the predictive value of SAF measurements were performed in selected patient populations [17, 20, 21]. SAF did predict cardiac mortality in people with diabetes [29] and in patients on chronic haemodialysis [30–32], and predicted the occurrence of cardiovascular events and mortality in individuals with peripheral vascular disease [33]. In addition, SAF predicted the subsequent need for limb amputation, which was independent of disease status, and proved to be additive to the predictive value of the Fontaine classification [34].

Our findings support the clinical utility of SAF to support risk assessment for cardiovascular disease and mortality, both in the general population and in people with type 2 diabetes. As suggested earlier [22], SAF measurement is relatively fast and non-invasive, and can therefore also be used outside a G.P. practice or hospital such as in pharmacies as a first estimate of risk. The present data also confirm that both current smoking and presence of cardiovascular disease are strong predictors for future CVD events and mortality, supporting aggressive intervention for smoking cessation as well as optimizing efforts to reduce cardiovascular disease burden. The fact that in the current study only 63% of people with known type 2 diabetes were using statins, and mean LDL-cholesterol was above the desired target suggests that improvements in cardiovascular risk factor intervention are desirable [35].

### Strengths and limitations

We presented data from a population-based study that included over 2300 participants with type 2 diabetes. The Lifelines Cohort Study has no follow-up data available on the use of new glucose-lowering or other medications or changes in medications, which could be used to confirm new CVD events and could influence the subsequent clinical course. New diagnosis of type 2 diabetes could only be based on a single blood glucose or HbA1c measurement. The current dataset of lifelines does not allow to perform survival analysis. The study included mainly participants from Western European background, and therefore our results may not be generalizable to other ethnic populations. Data on the exact cause of death may be helpful in further refining the predictive power of SAF.

## Conclusions

In conclusion, we showed that skin autofluorescence measurements significantly predicted new CVD events and mortality in people with type 2 diabetes independently of conventional cardiovascular risk factors. The Lifelines study is still ongoing, and a longer follow-up of its participants will expand the current evaluations and allow further validation.

## Supplementary Information

**Additional file 1: Figure S1.** Flow chart indicating the disposition of participants.

**Additional file 2: Figure S2.** Incidence of CVD events and death according to age.

**Additional file 3: Table S1.** Univariable and multivariable logistic regression analyses for the separate primary outcomes (CVD or death) at a median of 3.7 year follow-up.

**Additional file 4: Table S2.** Univariable and multivariable logistic regression analyses for the composite primary outcome (CVD or death) at a median of 3.7 years’ follow-up of people with baseline type 2 diabetes without clinically-manifest CVD.

## Data Availability

The manuscript is based on data from the Lifelines Cohort Study. Lifelines adheres to standards for data availability, and allows access for reproducibility of the study results. The data catalogue of Lifelines is publicly accessible at www.lifelines.nl. The dataset supporting the conclusions of this article is available through the Lifelines organisation (e-mail: research@lifelines.nl). For data access, a fee is required.

## Abbreviations

AGE: Advanced glycation endproducts
AU: Arbitrary units
BMI: Body mass index
CVD: Cardiovascular disease
DBP: Diastolic BP
eGFR: Estimated glomerular filtration rate
HbA1c: Glycated haemoglobin
HR: Heart rate
IQR: Interquartile range
SAF: Skin autofluorescence
SBP: Systolic BP
T2D: Type 2 diabetes
