# Supplementary figures and images for: Skin autofluorescence predicts new cardiovascular disease and mortality in people with type 2 diabetes
Source: BMC Endocr Disord. 2021 Jan 12;21:14. doi: 10.1186/s12902-020-00676-4 (PMC7802158; doi:10.1186/s12902-020-00676-4)

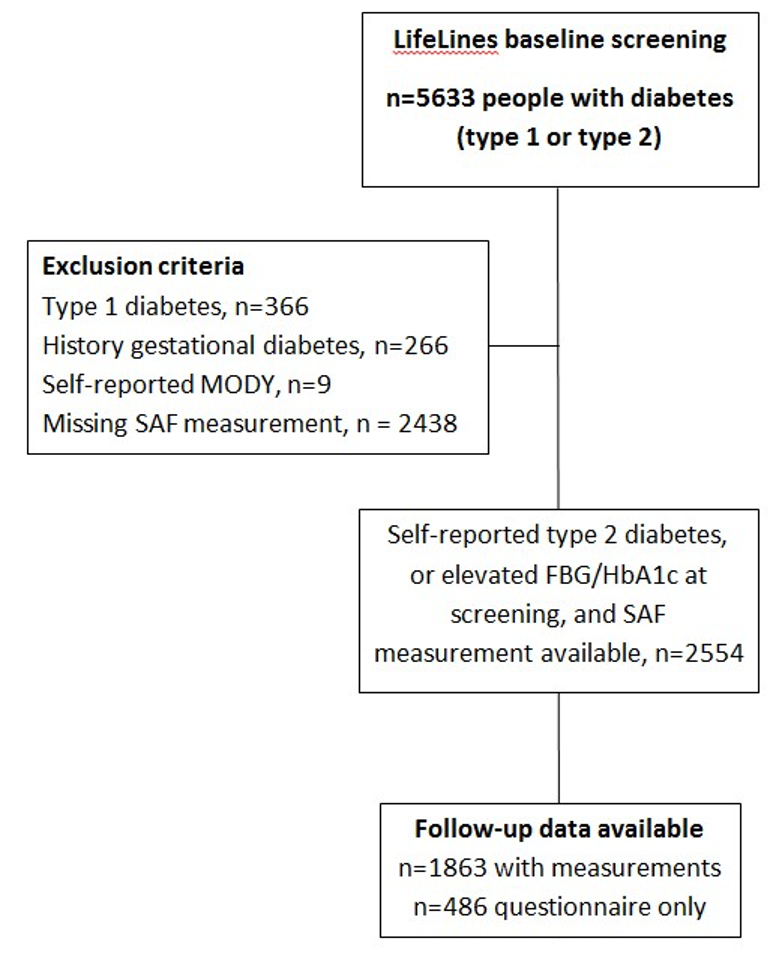

Supplement: Supplementary file 1 — Additional file 1: Figure S1. Flow chart indicating the disposition of participants. [file 12902_2020_676_MOESM1_ESM.png]

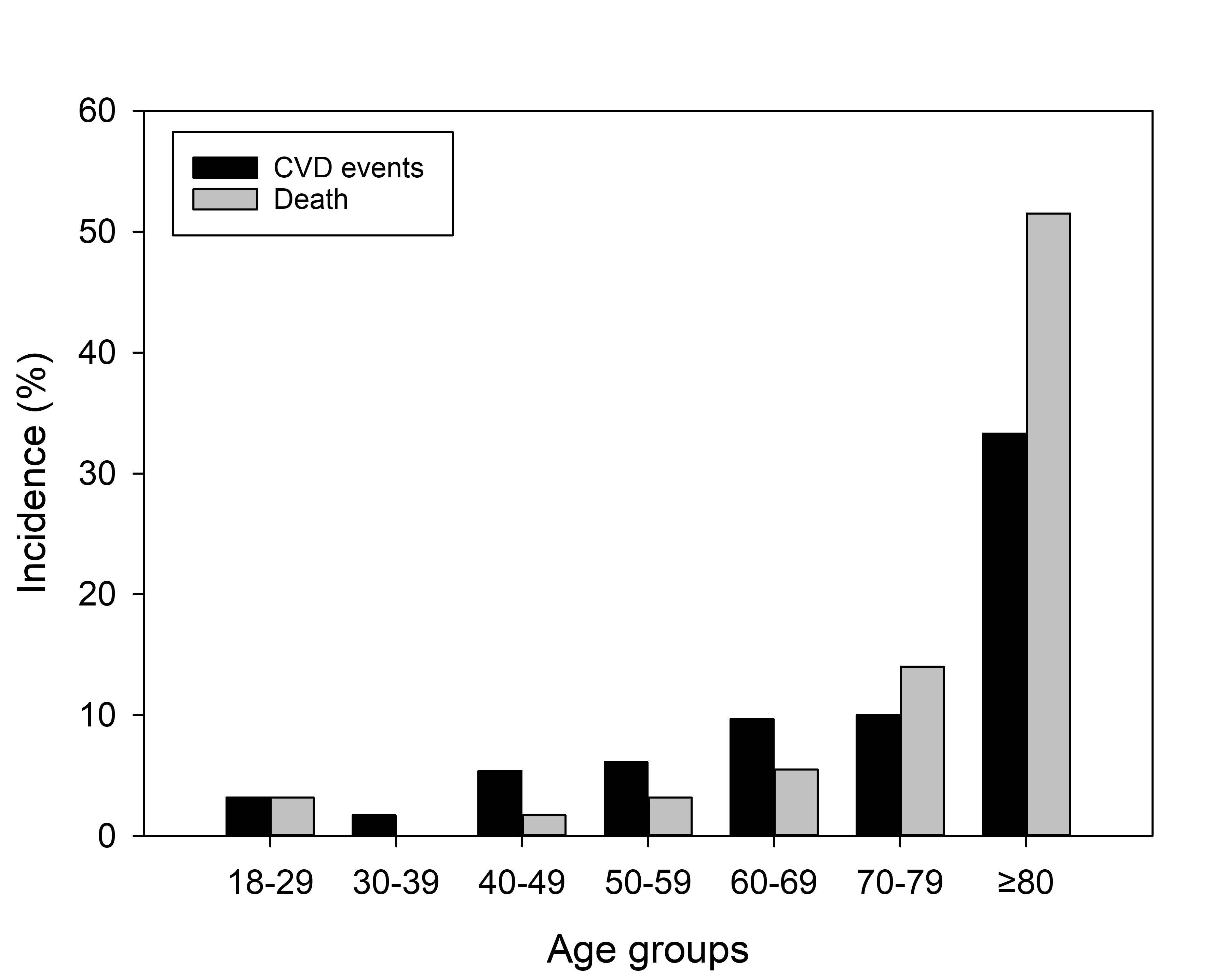

Supplement: Supplementary file 2 — Additional file 2: Figure S2. Incidence of CVD events and death according to age. [file 12902_2020_676_MOESM2_ESM.png]
