# Supplementary material for: Skin autofluorescence predicts new cardiovascular disease and mortality in people with type 2 diabetes
Source: BMC Endocr Disord. 2021 Jan 12;21:14. doi: 10.1186/s12902-020-00676-4 (PMC7802158; doi:10.1186/s12902-020-00676-4)
Supplement: Supplementary file 3 — Additional file 3: Table S1. Univariable and multivariable logistic regression analyses for the separate primary outcomes (CVD or death) at a median of 3.7 year follow-up. [file 12902_2020_676_MOESM3_ESM.docx]

**Additional file 3, Table 1.** Univariable and multivariable logistic regression analyses for the separate primary outcomes (CVD or death) at a median of 3.7 year follow-up

| Analysis | New CVD | | Death | |
| --- | --- | --- | --- | --- |
| Univariable analysis | OR (95% CI) | P-value | OR (95% CI) | P-value |
| SAF (AU) | 2.05 (1.61-2.62) | 8.9×10^−9^ | 2.98 (2.25, 3.94) | 2.6×10^−14^ |
| Age (years) | 1.04 (1.03, 1.06) | 8.0×10^−10^ | 1.10 (1.08, 1.12) | 2.4×10^−22^ |
| Male sex (y/n) | 1.45 (1.07, 1.96) | 0.015 | 1.54 (1.08, 2.19) | 0.018 |
| BMI (kg/m^2^) | 1.00 (0.97, 1.03) | 0.944 | 1.00 (0.97, 1.04) | 0.825 |
| Waist (cm) | 1.01 (1.00, 1.02) | 0.076 | 1.02 (1.00, 1.03) | 0.017 |
| Glucose (mmol/l) | 0.99 (0.92, 1.06) | 0.795 | 1.01 (0.93, 1.09) | 0.886 |
| HbA_1c_ (mmol/mol) | 1.01 (1.00, 1.02) | 0.109 | 1.00 (0.99, 1.02) | 0.425 |
| SBP (mmHg) | 1.00 (0.99, 1.00) | 0.285 | 1.00 (0.99, 1.01) | 0.450 |
| DBP (mmHg) | 0.97 (0.96, 0.99) | 0.001 | 0.97 (0.95, 0.99) | 0.002 |
| Heart rate (bpm) | 0.98 (0.97, 0.99) | 0.001 | 1.00 (0.98-1.01) | 0.957 |
| Cholesterol (mmol/l) | 0.92 (0.81, 1.05) | 0.236 | 0.88 (0.75, 1.02) | 0.095 |
| Triacylglycerol (mmol/l) | 1.02 (0.91, 1.15) | 0.740 | 0.93 (0.78, 1.09) | 0.350 |
| eGFR (ml/min/1.73m^2^) | 0.98 (0.97, 0.99) | 5.6×10^−5^ | 0.96 (0.95, 0.97) | 3.2×10^−13^ |
| Former smoking (y/n) | 1.62 (1.20, 2.18) | 0.002 | 0.99 (0.70, 1.40) | 0.957 |
| Current smoking (y/n) | 1.16 (0.81, 1.67) | 0.418 | 1.10 (0.71, 1.70) | 0.666 |
| Statin (y/n) | 1.67 (1.25, 2.25) | 0.001 | 1.19 (0.84, 1.68) | 0.328 |
| BP-lowering therapy (y/n) | 2.34 (1.71, 3.22) | 1.4×10^−7^ | 2.25 (1.55, 3.26) | 2.0×10^−5^ |
| Baseline CVD (y/n) | 7.85 (5.71, 10.8) | 1.2×10^−36^ | 3.85 (2.62, 5.66) | 7.2×10^−12^ |
| Multivariable model |  |  |  |  |
| SAF (AU) | 1.22 (0.91, 1.66) | 0.188 | 1.52 (1.09, 2.11) | 0.013 |
| Age (years) | 1.02 (1.00, 1.05) | 0.044 | 1.09 (1.06, 1.12) | 1.7×10^−10^ |
| Male sex (y/n) | 0.93 (0.62, 1.38) | 0.717 | 1.61 (0.99, 2.63) | 0.057 |
| BMI (kg/m^2^) | 0.95 (0.89, 1.02) | 0.180 | 0.97 (0.90, 1.05) | 0.476 |
| Waist (cm) | 1.02 (1.00, 1.05) | 0.087 | 1.03 (1.00, 1.06) | 0.092 |
| Glucose (mmol/l) | 0.96 (0.86, 1.06) | 0.405 | 1.06 (0.93, 1.20) | 0.420 |
| HbA_1c_ (mmol/mol) | 1.02 (1.00, 1.04) | 0.060 | 1.00 (0.97, 1.02) | 0.901 |
| SBP (mmHg) | 1.00 (0.99, 1.01) | 0.865 | 0.99 (0.97, 1.00) | 0.045 |
| DBP (mmHg) | 0.99 (0.98, 1.01) | 0.241 | 0.99 (0.96, 1.02) | 0.561 |
| Heart rate (bpm) | 1.00 (0.98, 1.01) | 0.630 | 1.03 (1.01, 1.04) | 0.002 |
| Cholesterol (mmol/l) | 1.20 (1.01, 1.42) | 0.043 | 0.93 (0.74, 1.16) | 0.502 |
| Triacylglycerol (mmol/l) | 1.01 (0.87, 1.18) | 0.863 | 1.01 (0.83, 1.23) | 0.907 |
| eGFR (ml/min/1.73m^2^) | 1.00 (0.99, 1.02) | 0.543 | 1.00 (0.98, 1.01) | 0.676 |
| Former smoking (y/n) | 1.39 (0.93, 2.08) | 0.111 | 0.58 (0.37, 0.91) | 0.018 |
| Current smoking (y/n) | 1.93 (1.17, 3.20) | 0.010 | 1.28 (0.72, 2.27) | 0.394 |
| Statin (y/n) | 0.94 (0.62, 1.41) | 0.747 | 0.56 (0.34, 0.91) | 0.019 |
| BP-lowering therapy (y/n) | 1.28 (0.86, 1.89)_ | 0.222 | 1.29 (0.82, 2.03) | 0.269 |
| Baseline CVD (y/n) | 6.91 (4.63, 10.3) | 3.2×10^−21^ | 2.31 (1.41, 3.77) | 0.001 |
|  |  |  |  |  |
| Multivariable model 2 |  |  |  |  |
| SAF (AU) | 1.28 (0.96, 1.72) | 0.096 | 1.54 (1.12, 2.13) | 0.008 |
| Age (years) | 1.03 (1.01, 1.04) | 0.002 | 1.10 (1.07, 1.12) | 1.3×10^−16^ |
| Waist (cm) | 1.01 (1.00, 1.02) | 0.110 | 1.03 (1.01, 1.04) | 3.6×10^−4^ |
| SBP (mmHg) | 0.99 (0.98, 1.00) | 0.170 | 0.99 (0.98,1.00) | 0.009 |
| Current smoking (y/n) | 1.59 (1.06, 2.39) | 0.024 | 1.96 (1.21, 3.19) | 0.007 |
| Statin (y/n) | 0.82 (0.58, 1.16) | 0.275 | 0.64 (0.43, 0.95) | 0.027 |
| Baseline CVD (y/n) | 6.77 (4.68, 9.78) | 2.3×10^−24^ | 2.34 (1.48, 3.68) | 2.5×10^−4^ |

Baseline risk factors were used to predict the median 3.7 year risk of the composite outcome of new CVD events and death. In both models, the same variables were selected as in Table 3.

SAF, age, glucose, HbA1c, waist circumference, systolic and diastolic BP, HR, cholesterol, triacylglycerol and eGFR were defined as continuous variables. Male sex, current smoking (vs never smoking), statin use, use of BP-lowering therapy and baseline CVD were defined as categorical variables

DBP, diastolic BP; SBP, systolic BP; HR, heart rate; y/n, yes/no
