# Supplementary material for: Skin autofluorescence predicts new cardiovascular disease and mortality in people with type 2 diabetes
Source: BMC Endocr Disord. 2021 Jan 12;21:14. doi: 10.1186/s12902-020-00676-4 (PMC7802158; doi:10.1186/s12902-020-00676-4)
Supplement: Supplementary file 4 — Additional file 4: Table S2. Univariable and multivariable logistic regression analyses for the composite primary outcome (CVD or death) at a median of 3.7 years’ follow-up of people with baseline type 2 diabetes without clinically-manifest CVD. [file 12902_2020_676_MOESM4_ESM.docx]

**Additional file 4, Table 2.** Univariable and multivariable logistic regression analyses for the composite primary outcome (CVD or death) at a median of 3.7 years’ follow-up of people with baseline type 2 diabetes **without** clinically-manifest CVD

| Analysis | n | OR | 95% CI | P-value |
| --- | --- | --- | --- | --- |
| Univariable |  |  |  |  |
| SAF (AU) | 2071 | 2.64 | 2.04-3.43 | 2.5×10^−13^ |
| Age (years) | 2071 | 1.06 | 1.04-1.08 | 1.3×10^−15^ |
| Male sex (y/n) | 2071 | 1.17 | 0.87-1.57 | 0.289 |
| BMI (kg/m^2^) | 2069 | 0.99 | 0.97-1.02 | 0.675 |
| Waist circumference (cm) | 2069 | 1.01 | 1.00-1.02 | 0.174 |
| Glucose (mmol/l) | 2065 | 1.02 | 0.96-1.09 | 0.509 |
| HbA_1c_ (mmol/mol) | 2059 | 1.01 | 1.00-1.02 | 0.037 |
| SBP (mmHg) | 2065 | 1.00 | 0.99-1.01 | 0.839 |
| DBP (mmHg) | 2065 | 0.99 | 0.97-1.00 | 0.093 |
| HR (/min) | 2065 | 1.00 | 0.99-1.01 | 0.958 |
| Cholesterol (mmol/l) | 2065 | 1.14 | 1.02-1.29 | 0.027 |
| Triacylglycerol (mmol/l) | 2065 | 1.03 | 0.92-1.15 | 0.647 |
| eGFR (ml/min/1.73m^2^) | 2066 | 0.98 | 0.97-0.99 | 5.0×10^−7^ |
| Former smoking (y/n) | 2058 | 0.96 | 0.71-1.29 | 0.789 |
| Current smoking (y/n) | 2058 | 1.35 | 0.95-1.91 | 0.092 |
| Statin (y/n) | 2071 | 0.80 | 0.59-1.08 | 0.149 |
| BP-lowering therapy (y/n) | 2071 | 1.42 | 1.06-1.91 | 0.019 |
|  |  |  |  |  |
| Multivariable model 1 | 2038 |  |  |  |
| SAF (AU) |  | 1.65 | 1.22-2.24 | 0.001 |
| Age (years) |  | 1.07 | 1.04-1.09 | 1.9×10^−8^ |
| Male sex (y/n) |  | 1.16 | 0.79-1.69 | 0.448 |
| BMI (kg/m^2^) |  | 0.97 | 0.91-1.03 | 0.330 |
| Waist (cm) |  | 1.02 | 1.00-1.05 | 0.085 |
| Glucose (mmol/l) |  | 0.99 | 0.89-1.10 | 0.821 |
| HbA1c (mmol/mol) |  | 1.01 | 0.99-1.03 | 0.208 |
| SBP (mmHg) |  | 0.99 | 0.98-1.00 | 0.047 |
| DBP (mmHg) |  | 1.00 | 0.98-1.02 | 0.989 |
| HR (/min) |  | 1.01 | 1.00-1.02 | 0.114 |
| Cholesterol (mmol/l) |  | 1.10 | 0.94-1.30 | 0.240 |
| Triacylglycerol (mmol/l) |  | 1.02 | 0.88-1.17 | 0.802 |
| eGFR (ml/min/1.73m^2^) |  | 1.00 | 0.99-1.02 | 0.750 |
| Former smoking (y/n) |  | 0.88 | 0.61-1.26 | 0.486 |
| Current smoking (y/n) |  | 1.51 | 0.96-2.37 | 0.072 |
| Statin (y/n) |  | 0.67 | 0.46-0.99 | 0.046 |
| BP-lowering therapy (y/n) |  | 1.19 | 0.85-1.68 | 0.310 |
|  |  |  |  |  |
| Multivariable model 2 | 2050 |  |  |  |
| SAF (AU) |  | 1.74 | 1.29-2.34 | 2.5×10^−4^ |
| Age (years) |  | 1.06 | 1.04-1.08 | 1.5×10^−12^ |
| Waist (cm) |  | 1.02 | 1.00-1.03 | 0.012 |
| SBP (mmHg) |  | 0.99 | 0.98-1.00 | 0.021 |
| Current smoking (y/n) |  | 1.70 | 1.16-2.49 | 0.006 |
| Statin (y/n) |  | 0.62 | 0.45-0.85 | 0.003 |

Baseline risk factors were used to predict the median 3.7 year risk of the composite outcome of CVD and death

SAF, age, glucose, HbA_1c_, waist circumference, systolic and diastolic BP, HR, cholesterol, triacylglycerol, eGFR and were defined as continuous variables. Male sex, current smoking (vs never smoking), statin use, use of BP-lowering therapy were defined as categorical variables

DBP, diastolic BP; SBP, systolic BP; HR, heart rate; y/n, yes/no
